# The microbiome of African penguins (*Spheniscus demersus*) under managed care resembles that of wild marine mammals and birds

**DOI:** 10.1038/s41598-023-43899-w

**Published:** 2023-10-04

**Authors:** Ana G. Clavere Graciette, Lisa A. Hoopes, Tonya Clauss, Frank J. Stewart, Zoe A. Pratte

**Affiliations:** 1 School of Biological Sciences, Center for Microbial Dynamics and Infection, Georgia Institute of Technology, Atlanta, GA USA; 2 Department of Microbiology & Cell Biology, Montana State University, Bozeman, MT USA; 3 Georgia Aquarium, Atlanta, GA USA

**Keywords:** Microbiome, Microbial ecology

## Abstract

Animals under managed care in zoos and aquariums are ideal surrogate study subjects for endangered species that are difficult to obtain in the wild. We compared the fecal and oral microbiomes of healthy, managed African penguins (*Spheniscus demersus*) to those of other domestic and wild vertebrate hosts to determine how host identity, diet, and environment shape the penguin microbiome. The African penguin oral microbiome was more similar to that of piscivorous marine mammals, suggesting that diet and a marine environment together play a strong role in shaping the oral microbiome. Conversely, the penguin cloaca/fecal microbiome was more similar to that of other birds, suggesting that host phylogeny plays a significant role in shaping the gut microbiome. Although the penguins were born under managed care, they had a gut microbiome more similar to that of wild bird species compared to domesticated (factory-farmed) birds, suggesting that the managed care environment and diet resemble those experienced by wild birds. Finally, the microbiome composition at external body sites was broadly similar to that of the habitat, suggesting sharing of microbes between animals and their environment. Future studies should link these results to microbial functional capacity and host health, which will help inform conservation efforts.

## Introduction

It is now widely accepted that host-associated microbes influence vertebrate digestion, organ development, immune system function, behavior, protection against pathogens, and overall health^1–3^. Gut microbial communities can affect processes that extend beyond the individual, such as mating and reproduction, or the spread of disease or antibiotic resistance^4,5^. Because of the important role microbes play in animal development and health, determining the diversity, function, variability, and influencing factors of the microbiome across animal hosts has emerged as a research priority^6–9^.

While host identity appears to strongly influence an animal's gut microbiome composition^10–13^, the establishment and composition of the vertebrate microbiota are also influenced by an array of other factors, including environmental condition, social interaction, diet, age, sex, gut physiology, and host health status^14–19^. For example, environmental factors such as the nesting environment can shape a bird’s microbiome and may be particularly influential in young birds^10,20–22^. However, the relative effects of these factors are not known for most animals and may vary depending on species, developmental stage, life history characteristics, and geographical location.

Despite strong evidence for the importance of microbiomes in animal health, microbiome studies targeting wild and endangered animals remain uncommon. Animals under managed care in zoos and aquaria are ideal study surrogates for wild populations that are understudied due to logistical challenges, permit restrictions, and rarity. Additionally, a highly controlled managed care environment reduces the number of confounding variables usually present in the wild. Thus, endangered animals under managed care are ideal research targets. Understanding how a built environment shapes the microbiome of its residents can also inform management strategies that seek to optimize animal health, including the health of animals in zoos, aquariums, or research facilities.

The African penguin (*Spheniscus demersus*) is listed as endangered on the IUCN Red List. Its populations have decreased by ~ 70% in the past 50 years and are still decreasing despite active conservation efforts^23^. Disease control has been identified as an important focus for the stabilization and recovery of African penguin populations^24^. However, the implementation of such strategies requires a baseline knowledge of the African penguin microbiome. Sampling *Spheniscus demersus* microbiomes from wild birds presents physical challenges that can be overcome to some extent by studying birds under managed care in zoos and aquariums. In this study, we hypothesized that the African Penguin microbiome would be strongly influenced by diet, physiology, and environment. We hypothesized that the external surfaces of the African penguin are primarily influenced by interactions with exhibit water and surfaces, while internal body sites such as the mouth and feces are primarily influenced by diet or physiology. We predicted microbiome variation to be manifest both in the proportional representation of microbes, as well as in the number of microbial types (alpha diversity). The latter is sometimes but not always predictive of functional niche breadth in a community and in some but not all host-associated microbiomes co-varies with both host health and microbiome stability. To test these hypotheses, we characterized the microbiome of African penguins from Georgia Aquarium using 16S rRNA gene amplicon sequencing. To determine the influences of diet and environment on internal body locations, we focused primarily on the fecal microbiome, as well as the rarely characterized oral microbiome. In a meta-analysis, we compared the African penguin microbiome to that of other wild and domesticated vertebrates in order to identify the relative influences of host identity, diet, and environment on these two body sites. In addition, we compared the microbial communities associated with the skin, uropygial (preening) gland, and brood pouch, to fallen feathers and other environmental samples from the penguin exhibit to assess the extent to which body surface microbiomes shape or are shaped by those of the surrounding habitat.

## Materials and methods

### Sample collection

Samples were obtained between November 2018 and March 2019 from various body sites (described below) of 36 African penguins (*Spheniscus demersus*) under managed care at Georgia Aquarium (Atlanta, GA, USA). All sampled penguins were born under managed care either at Georgia Aquarium or another zoological institution before being transferred to Georgia Aquarium (Supplementary Table 1). At the time of sampling, Georgia Aquarium housed approximately 55 penguins, with the number of individuals occupying the exhibit fluctuating as chicks were born and animals were moved on and off exhibit. Penguins were maintained in an indoor, 16,500-gallon exhibit that contained an artificial rocky environment and 3 basins filled with artificial seawater 0.5–1.5 m deep. All basins were connected and supplied from the same aquarium life support system. The water from the entire exhibit was turned over every 37 min using a filtration system composed of two vertical sand filters, a protein fractionator, an ozone contactor, and a deaeration tower. The water temperature was maintained at 17 °C using a titanium plate heat exchanger, and penguins were fed a mixed piscivorous diet (capelin (*Mallotus villosus*), Pacific herring (*Clupea pallasii*), squid (*Loligo *sp.), night smelt (*Spirinchus starksi*), and silversides (*Menidia menidia*)) ad libitum twice a day.

Microbiome samples were collected opportunistically while the birds were removed from the exhibit for routine annual health exams or as needed to address minor medical concerns. No birds were sampled more than once. All body site samples were collected by gently rubbing sterile swabs along the cloaca, oral cavity (near the choana), brood pouch, uropygial (preening) gland, leg skin, or back skin, accumulating material over the entire surface of the swab. For skin samples, feathers were parted to access the skin. Fecal samples and feather samples were also collected opportunistically when they fell to the floor during examination—these were considered “environmental” samples as they were no longer a part of the penguin body. Samples were immediately preserved in an RNA/DNA stabilizing buffer (25 mM sodium citrate, 10 mM EDTA and 70 g ammonium sulfate per 100 ml solution, pH 5.2) and frozen at – 80 °C until further processing. Not all body sites were sampled from all penguins (Supplementary Table 1). Sample collection was approved by the IACUC ethics committee at Georgia Institute of Technology, under protocol A100174. All penguin samples were collected by veterinary staff at the aquarium in accordance with the relevant guidelines and regulations, in accordance with ARRIVE guidelines.

Swabs were also collected from surfaces within the exhibit, including dry rocks, wet rocks, the waterline of the exhibit, fresh and dry guano, and three different nesting niches. Water microbiome samples from the exhibit basin were collected by filtering basin water through a 0.2 µm Sterivex filter, as described in our prior work (e.g.,^25^). Penguin food microbiome samples were also collected by rubbing a sterile swab on the external surface and into the cloaca of fish and other mollusks contained in a bucket of food to be delivered to the penguins. All environmental samples (water, surface and food swabs) were collected at a single time point in July 2018. Samples were immediately preserved in RNA/DNA stabilizing buffer, and frozen at – 80 °C until further processing. A summary of all samples is included in Tables 1 and S1.

**Table 1:** Summary of samples from the African penguins (*Spheniscus demersus*) of Georgia Aquarium.

Sample type	Number of samples	Number of ASVs
Body sites		
Cloaca	33	815
Oral cavity	35	650
Brood pouch	18	1145
Uropygial (preening) gland	18	1067
Back skin	9	812
Leg skin	20	1122
Environment and opportunistic samples		
Feather*	3	444
Fecal*	2	223
Dry rock	2	536
Wet rock	2	262
Water	2	824
Shoreline	3	538
Nest	4	416
Dry guano**	2	200
Food	8	595

The number of samples per body site or location and the total number of amplicon sequence variants (ASVs) associated with each sample type are given.

*Opportunistically collected from the floor during routine veterinary examinations.

**Dry fecal matter collected from rocks in the exhibit.

### DNA extraction and sequencing

Total DNA was extracted from swabs using the PowerSoil DNA extraction kit (QIAGEN, Location, USA). Swabs were placed directly into PowerBead tubes and extracted according to the manufacturer’s instructions. For each kit, an extraction blank (sterile swabs without biomass) was processed following the same procedures, resulting in four extraction blanks total. DNA from Sterivex filters was extracted following the protocol described in^25^. For each sample, the V3–V4 region of the 16S rRNA gene was amplified by PCR using primers F515 and R806 (^26^ with modifications according to^27,28^), each appended with barcodes and Illumina-specific adapters^29^. Reaction mixtures included 2–5 μl DNA template, 12.5 μl Hot Start Taq PCR MasterMix (VWR), 0.25 μl (each) forward and reverse primers (20 μm), and 0.5 μl bovine serum albumin (BSA) (20 mg/ml; New England BioLabs Inc), and brought up to 25 μl with sterile nuclease free water. PCR conditions included an initial 1 min denaturation at 94 °C, followed by 30 cycles of denaturation at 94 °C (1 min), primer annealing at 55 °C (2 min), and extension at 72 °C (90 s) and then a final extension at 72 °C for 10 min. Amplicon libraries were purified using Diffinity RapidTip PCR purification tips (Diffinity Genomics, NY), quantified fluorometrically on a Qubit (Life Technologies), and pooled at equimolar concentrations. Amplicons were sequenced on an Illumina MiSeq using a V2 500-cycle kit (250 × 250 bp) with 5% PhiX to increase read diversity.

### Illumina data processing

Raw reads were quality checked using the DADA2 R-package^30^ and QIIME 2 2019.4^31^. All forward reads were demultiplexed, quality filtered and trimmed to 175 bp following the DADA2 pipeline from^30^, while reverse reads were discarded due to lower quality. Taxonomy was assigned to amplicon sequence variants (ASVs) using the SILVA-132 database. The resulting representative sequences, taxonomy and ASV tables were imported into QIIME 2 2019.4^31^. Sequences classified as *Chloroplast* and *Eukaryota* were removed from the dataset. In QIIME2, all ASVs were aligned with Mafft^32^, via q2‐alignment, and used to construct a phylogeny with fasttree2^33^, via q2‐phylogeny. All penguin and oral meta-analysis samples were rarefied (subsampled without replacement) to 2500 reads, and the fecal/cloaca meta-analysis samples were rarefied to 2000 sequences. All sequence data are publicly available in NCBI's SRA database under Project Number PRJNA757721 and PRJNA757724.

### Meta-analysis data processing

To assess the relative influence of host identity, diet, and environment in shaping the oral and cloaca penguin microbiomes, we compared the African penguin oral and cloacal datasets to cloacal or fecal, and oral microbiomes of other domestic and wild birds, reptiles and mammals (including marine mammals). Datasets included in the meta-analyses were selected on the criteria that they provide informative comparisons to the African penguin, targeted the V3–V4 region of the 16S rRNA gene, and were generated using either 454 or Illumina sequencing technologies. A summary of these datasets is in Table 2. All datasets were trimmed to retain the same V3–V4 region amplified in this study. Datasets sequenced using Illumina technology were then pooled and processed independently through DADA2 using the same methods as above. Datasets sequenced using 454 technology were processed separately, with parameters adjusted in the DADA2 algorithm to deal with such data by modifying denoising parameters (HOMOPOLYMER_GAP_PENALTY = − 1, BAND_SIZE = 32) and filtering sequences by maximum length^30^. All ASV tables issued from Illumina and 454 sequencing technologies were merged and taxonomy was assigned following the same method as described above.

**Table 2:** Sample metadata and number of associated amplicon sequence variants (ASVs) for the oral and fecal microbiome meta-analyses.

Host species	Scientific name	Origin of the data and sequencing technology	Status	Types of samples	Number of fecal/cloaca samples	Number of fecal/cloaca ASVs	Number of oral samples	Number of oral ASVs
African penguin	*Spheniscus demersus*	The present study MiSeq (Illum.)	Managed care	Cloaca and oral	33	700	35	625
Barn swallow	*Hirundo rustica*	^54^ 454 (Roche)	Wild	Cloaca	8	463	Not sampled	Not sampled
Great tit	*Parus major*	^12^ MiSeq (Illum.)	Wild	Feces and oral	17	1240	20	1711
Broiler	*Gallus gallus domesticus*	^7^ 454 (Roche)	Domestic	Feces	27	1480	Not sampled	Not sampled
Egg laying hen	*Gallus gallus domesticus*	^7^ 454 (Roche)	Domestic	Feces	15	1738	Not sampled	Not sampled
Crocodile lizard	*Shinisaurus crocodilurus*	^16^ HiSeq (Illum.)	Managed care and wild	Cloaca	16	1528	Not sampled	Not sampled
Komodo dragon	*Varanus komodoensis*	^55^ HiSeq (Illum.)	Managed care	Feces and oral	46	1829	26	3912
California sea lion	*Zalophus californianus*	^56^ 454 (Life Sci.)	Managed care	Cloaca and oral	8	196	7	125
Bottlenose dolphin	*Tursiops truncatus*	^56^ 454 (Life Sci.)	Managed care and wild	Cloaca and oral	15	119	10	392
Human	*Homo sapiens*	^57^ Illu. & 454	NA	Feces and oral	41	1877	26	4956
Cooper's hawk	*Accipiter cooperii*	^58^ MiSeq (Illum.)	Wild	Oral	Not sampled	Not sampled	30	298
Mangrove monitor	*Varanus indicus*	^55^ HiSeq (Illum.)	Managed care	Oral	Not sampled	Not sampled	1	120
Gray's monitor lizard	*Varanus olivaceus*	^55^ HiSeq (Illum.)	Managed care	Oral	Not sampled	Not sampled	2	213
Black roughneck monitor	*Varanus rudicollis*	^55^ HiSeq (Illum.)	Managed care	Oral	Not sampled	Not sampled	1	91
Prairie rattlesnake	*Crotalus viridis*	^55^ HiSeq (Illum.)	Managed care	Oral	Not sampled	Not sampled	4	302

The datasets included in the meta-analyses were selected for their informative comparisons to the African penguin. Few studies have compared the oral microbiome across animals, potentially due to a lack of oral datasets from non-model taxa. We therefore characterized the oral microbiome of the African penguin relative to that of phylogenetically and ecologically divergent vertebrates for which oral microbiome data were publicly available. It should be noted that not all studies selected for the oral and fecal meta-analyses included both oral and fecal microbiomes from all animals.

### Statistical analysis

Statistical analyses were conducted through QIIME2^31^ to test for differences between penguin body sites and environmental samples, and between different animal classes (reptiles, birds, and mammals) and penguins when focusing on the oral and cloaca/fecal meta-analyses. A Kruskal–Wallis test was used to assess differences in alpha diversity (observed ASVs, and Shannon diversity index) and a PERMANOVA test was used to assess differences in beta diversity (weighted UniFrac). All analyses were performed using the rarefied ASV tables. Weighted Unifrac dissimilarity matrices were used to conduct Principle Coordinate Analysis (PCoA) using Primer-e v.7 (PRIMER-E Ltd, United Kingdom).

### Ethical approval

This study is approved by the IACUC ethics committee at Georgia Institute of Technology, under protocol A100174. This study is in accordance with relevant guidelines and regulations.

## Results

16S rRNA gene sequence data were generated for 176 penguin samples from various body sites including the cloaca, mouth, skin, brood pouch, and uropygial (preening) gland, as well as environmental samples including the exhibit water, surfaces in the exhibit, and the food (Supplementary Table S1). After quality filtering, trimming, and rarefaction, 161 samples constituting 3232 ASVs composed the final penguin dataset (Table 1). All negative controls and extraction blanks did not pass quality control, validating our quality control method. Meta-analyses were conducted comparing the penguin oral and cloaca/fecal microbiomes to those from other domestic and wild vertebrate species (Table 2). After quality filtering, trimming, and rarefaction, a total of 162 samples constituting 6522 ASVs were used for the oral meta-analysis, and 226 samples constituting 8795 ASVs were used for the fecal/cloaca meta-analysis (Table 2).

**Figure 1:**
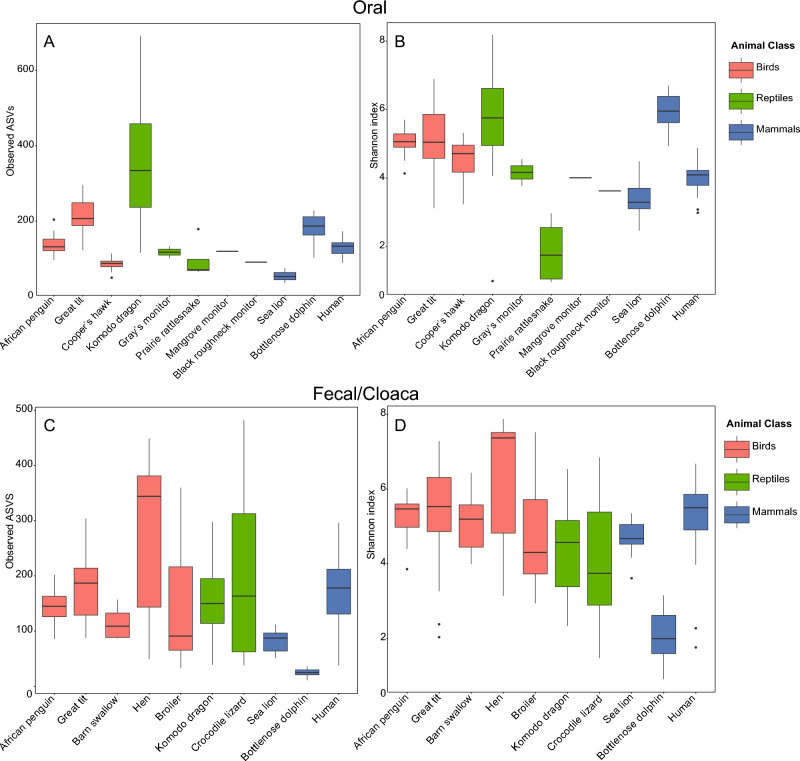
Standard box plots representing differences in observed ASVs (left column) and Shannon diversity index (right column) for the oral (top row) and fecal/cloaca (bottom row) microbiomes of various vertebrate hosts including birds, reptiles, and mammals. Significant differences in the Shannon diversity index are given in Supplementary Tables S4 and S6.

**Figure 2:**
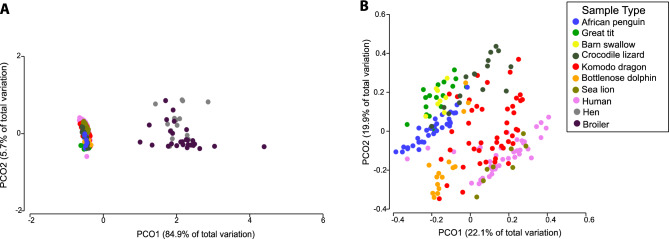
Principal coordinate analysis (PCoA) using weighted Unifrac distances between the fecal/cloaca microbiomes of various vertebrate hosts including wild and managed birds, reptiles, and mammals. (**A**) Includes poultry (hen and broiler) samples, (**B**) does not include poultry samples. Pairwise PERMANOVA tests are given in Supplementary Table S8.

Datasets included in the oral and fecal meta-analyses were selected because they allowed informative biological comparisons to the African penguin data and they were generated using methods and primer sets consistent with those used for our data. However, not all studies selected for these analyses included both oral and fecal microbiomes from all animals. These analyses revealed that the taxonomic composition and diversity indices for hen and broiler poultry samples were notably distinct compared to those of other vertebrates (Fig. 1; Fig. 2A). These datasets did not share several phyla present in all other bird hosts and were instead contained high representation (25–50%) by *Lactobacillus*, which represented < 3% of sequences in all other vertebrates (Supplementary Figures S1, S2). Poultry samples were therefore removed from statistical analyses.

### Penguin microbiomes from external body sites are similar to those of the environment

**Figure 3:**
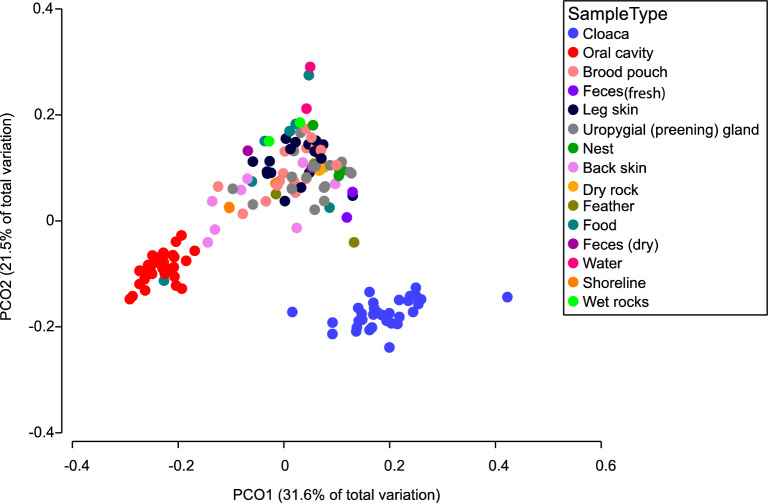
Principal coordinate analysis (PCoA) using weighted Unifrac distances between the microbiome of various African penguin (*Spheniscus demersus*) body sites and environmental samples of the penguin exhibit at Georgia Aquarium. Pairwise PERMANOVA tests are given in Supplementary Table S3.

When assessed using the Shannon diversity index, alpha diversity did not differ significantly between microbiomes of external body sites of the African penguin (brood pouch, preening gland, leg skin, and back skin) and those of environmental samples (Kruskal–Wallis p values > 0.05; Supplementary Table S2; Supplementary Figure S3). In contrast, microbiome taxonomic composition—beta diversity measured by weighted UniFrac distances—differed among sample types, with cloaca and oral samples differing significantly and clustering to the exclusion of samples from more external body sites (Fig. 3, Supplementary Table S3). While composition in some external body site samples differed significantly from that of environmental samples, external body site microbiomes were generally more similar to those of the surrounding habitat than to those of internal body sites (Fig. 3, Supplementary Table S3).

All external penguin and environmental samples were primarily dominated by *Proteobacteria*, *Firmicutes*, *Bacteroidetes*, and *Actinobacteria* (Supplementary Figure S4). *Actinomyces*, *Petrimonas*, *Campylobacter*, and *Fastidiosipila* were more abundant in the cloaca microbiome, while *Coenonia* and *Suttonella* were prominent in the oral cavity (Supplementary Figure S5). To assess possible routes of microbial exchange, we examined those taxa shared among penguin and environmental samples. Over 60% of ASVs in external body site samples were also detected in environmental samples, with ASVs of the genera *Psychrobacter* and *Oceanisphaera* being particularly common in both external body site and environmental samples (Supplementary Figure S3). In contrast, less than 45% of cloaca and oral ASVs were shared with environmental samples, and the fraction of unique ASVs within the oral and cloaca microbiomes was twice that of other body sites (Supplementary Table S4). Microbiome composition of African penguin body sites was not significantly influenced by metadata variables age, sex, time since molt, reproductive status, use of a nest, use of specific medications and supplements, or antiseptic treatment (Kruskal–Wallis test for alpha diversity and PERMANOVA for beta diversity—p > 0.05). However, these tests had low statistical power due to small sample size; further investigation is needed to evaluate the influence of these variables.

### The penguin oral microbiome is similar to that of marine mammals

**Figure 4:**
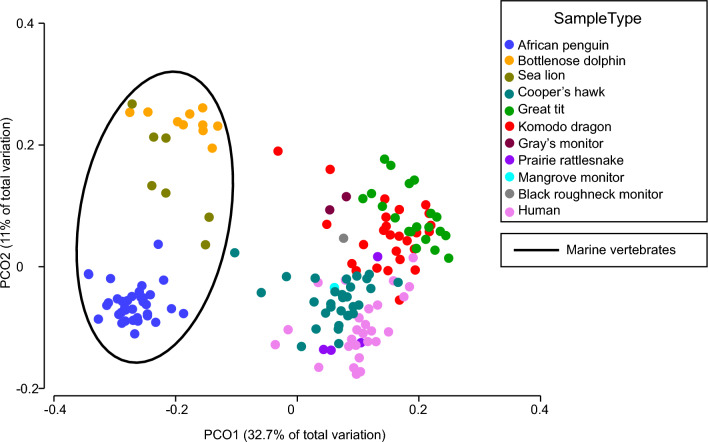
Principal coordinate analysis (PCoA) using weighted Unifrac distances between the oral microbiomes of various vertebrate hosts including wild and managed birds, reptiles, and mammals. Marine vertebrates are circled in black. Pairwise PERMANOVA tests are given in Supplementary Table S6.

**Figure 5:**
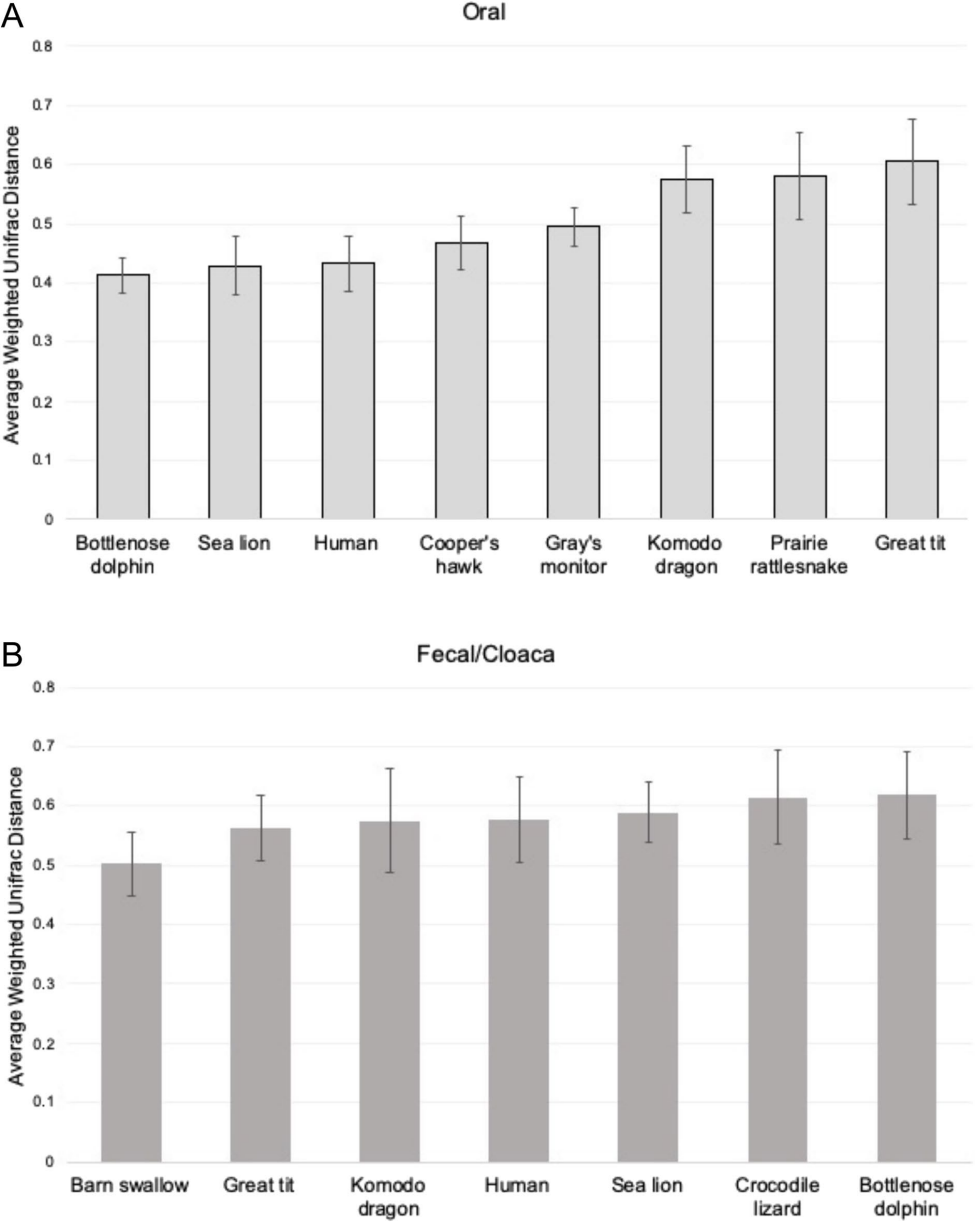
Average weighted Unifrac distances from the African penguin oral (panel **A**) and fecal/cloaca (panel **B**) microbiome. From left to right, average distances are arranged from smallest (more similar to the African penguin) to largest (least similar to the African Penguin). Individual samples and distances are plotted in Figs. 2 and 4. Error bars indicate standard deviation.

Few studies have compared the oral microbiome across animals, potentially due to a lack of oral datasets from non-model taxa. We therefore characterized the oral microbiome of the African penguin relative to that of phylogenetically and ecologically divergent vertebrates for which oral microbiome data (generated using the same primers as in our study) were publicly available. Based on the Shannon diversity index, alpha diversity in the African penguin oral microbiome was greater than that of the Cooper’s hawk (*Accipiter cooperii*), prairie rattlesnake (*Crotalus viridis*), sea lion (*Zalophus californiansus*), and human (*Homo sapiens*) oral microbiomes, but lower than that in the bottlenose dolphin (*Tursiops truncatus*) (Fig. 1; Supplementary Table S5). Based on weighted Unifrac distances, the taxonomic composition of the African penguin oral microbiome differed significantly from that of all other oral microbiomes (Supplementary Table S6). However, principal coordinate analysis showed oral microbiomes of penguins clustering with those of sea lions and dolphins, to the exclusion of oral microbiomes of non-marine vertebrates (Fig. 4); consistent with this clustering pattern, the average weighted UniFrac distance to penguin microbiomes was lowest for sea lion and dolphin datasets (Fig. 5). These trends suggest microbiome similarity in the oral cavities of marine animals.

At the phylum level, marine vertebrate oral microbiomes were again more similar to each other than to those of non-marine vertebrates and harbored the lowest relative abundances of *Actinobacteria* (< 2%) and *Firmicutes* (< 9%), but the highest relative abundance of *Bacteroidetes* (> 34%; Supplementary Figures S6, S7). Interestingly, the Gram-negative bacterial genus *Proteiniphilum* was found in the oral microbiome of all bird species but not in non-birds, though in low abundance (< 1%).

### The penguin fecal/cloaca microbiome is most similar to that of other birds

Alpha diversity in fecal/cloacal microbiomes—including richness and diversity estimated using the number of observed ASVs and the Shannon diversity index—varied among vertebrate species (Fig. 1). Based on the Shannon diversity index, the African penguin cloacal microbiome was significantly more diverse than that of the bottlenose dolphin and Komodo dragon (Fig. 1; Supplementary Table S7). Beta diversity analysis indicated that taxonomic composition in the penguin fecal/cloacal microbiome (based on weighted UniFrac distances) varied significantly from all other species (Supplementary Table S8). In contrast to the oral microbiome, the penguin fecal/cloacal microbiome was most similar to that of other birds, based on average weighted Unifrac distances (Fig. 2B; Fig. 5). The representation of Actinobacteria likely contributed to these differences, as Actinobacteria represented < 0.1% of sequences in mammal datasets, but > 13% of sequences in African penguins and wild birds (barn swallow, and great tit; Supplementary Figure S1). Other microbial taxa enriched in bird microbiomes include *Patescibacteria,* which was present almost exclusively in wild birds and the genera *Corynebactericeae* and *Catellicococcus,* which were uniquely shared by all bird fecal microbiomes, although these bacterial taxa represented a small fraction of the microbiome (< 1.5%) and were composed of different ASVs for each host species.

## Discussion

This study compared the microbiome of African penguins under managed care to the microbiome of their exhibit. This analysis showed the microbiome of external body sites to be similar to that of the exhibit, suggesting high connectivity between the birds and their managed, artificial habitat. We also conducted meta-analyses comparing penguin oral and fecal/cloacal microbiomes to that of other vertebrates both under managed care and free-living. The meta-analyses showed that the oral microbiome of African penguins was more similar to that of marine mammals, while the penguin fecal/cloaca microbiome was more similar to that of other wild birds (Fig. 2).

### The African penguin microbiome is strongly tied to the environment

Beta diversity analyses showed that the microbiomes of all external body sites were similar to those of the surrounding environment, and there were fewer statistically significant differences between the environmental samples and external body sites compared to internal body sites. Similarly, both environmental and external penguin samples were primarily dominated by the same phyla, including the bacterial genera *Oceanisphaera* and *Psychrobacter,* which have been associated with both planktonic^34,35^ and host-associated marine microbiomes^36,37^. Together, these trends suggest that the external African penguin microbiome is strongly influenced, and likely influences, the microbiome of immediate surrounding environment, with transfer of microbes likely routinely occurring between these body sites and the water and various surfaces of the managed care exhibit. This potential high connectivity suggests a need to keep the habitats of African penguins clean and free of pathogens and that routine monitoring of water and other habitat microbiomes may be useful in identifying microbes that are also in direct association with the animal surfaces. However, further work is needed to assess the extent to which microbial taxa are shared between host-associated and environmental niches under natural settings outside managed care.

### The penguin oral microbiome is similar to marine mammals

Although the African penguin oral microbiome differed significantly in composition from the oral microbiomes of all other animals tested, this microbiome was statistically most similar to that of other marine animals (Fig. 5). Moreover, all of these marine animals (the African penguin, sea lion, and bottlenose dolphin) are piscivores. Previous studies highlight the major role that diet plays in shaping the oral microbiota^38–40^, and suggest that diet is shaping the oral microbiome of these animals as well. However, it is difficult to disentangle the effects of diet versus other aspects specific to the marine environment (e.g., water column microbiome composition) that may also be shaping microbiome structure. It is likely that the marine lifestyle in general, combined with a piscivorous diet, are driving the similarity between the African penguin and marine mammal oral microbiomes, despite major differences in host anatomy and physiology.

Multiple interacting factors—including host physiology and oral anatomy, diet, and contact with the surrounding air/water—likely influence the diversity of the oral microbiome, notably as this body site niche is continually exposed to external components including air/water and food (in contrast to most sections of the gastrointestinal tract). Indeed, alpha diversity in the penguin oral microbiome, like other oral microbiomes analyzed here, is comparable to that of the cloaca/feces, suggesting a complex community potentially associated with diverse functional or spatial niches. The oral cavity and its associated community is the first component of the gastrointestinal tract, with its microbes potentially serving as inocula for other sections of the gastrointestinal system, underlining the importance of the oral system as part of the digestive process but also as a potential reservoir and vector of pathogens and disease^41–44^. For the African penguins in Georgia Aquarium, the most abundant ASVs (> 1%) unique to both the penguin oral and cloacal microbiome were not pathogenic and corresponded to taxonomic groups that are often found associated with the respiratory system or gastrointestinal tract of birds. These included microbes from the families *Cardiobacteriaceae*^45^*, Flavobacteriaceae*^9^, *Weeksellaceae*, *Moraxellaceae*^46^, and *Mycoplasmataceae*^47^*.* However, evaluating the function of these taxa in relation to host health is difficult, as none of these taxa could be classified to the genus level. Finally, while the penguin microbiome contained relatively high abundances (up to ~ 15%) of microbial species known to harbor some pathogenic strains (e.g., *Coenonia* in the oral microbiome), penguins from Georgia Aquarium were healthy and under constant veterinary supervision, which suggests that the ASVs recovered from these animals are likely nonpathogenic. These patterns establish a baseline for comparing oral microbiome signatures relative to changes in host health.

### The penguin fecal/cloaca microbiome is most similar to that of other birds

Based on previous research, phylogeny and diet are main factors shaping the microbiome of vertebrates^48–50^, with variation in the relative strength of these factors among host clades. In our fecal/cloacal meta-analysis, microbial taxonomic composition was more similar among wild birds compared to other mammals and reptiles (Fig. 5), suggesting that phylogeny plays a strong role in shaping the bird gut microbiome^12,13,51^. All birds, including the African penguin, had a fecal/cloacal microbiome composed of 4 major phyla including *Firmicutes*, *Bacteroidetes*, *Actinobacteria*, and *Proteobacteria*, which occurred at variable relative abundances among species and exhibited divergence in microbial richness and abundance when evaluated at finer taxonomic levels, as also seen in^9^. Two genera identified as *Corynebacteriaceae* and *Catellicococcus* were unique to bird fecal microbiomes and have been previously reported as common in the bird gastrointestinal tract^52,53^. Unlike the oral cavity, the African penguin cloaca microbiome was more similar to that of other wild birds (barn swallow and great tit), highlighting the effect of phylogeny as a main driver of microbiome structure in bird guts^12,13,51^.

Interestingly, the fecal microbiome of factory-farmed poultry was very different from that of any other vertebrates, including all birds, and was highly dominated by *Lactobacillus*. This suggests that while phylogeny plays a major role in structuring the bird gut microbiome, highly controlled environments such as those used in commercial farming over multiple generations and with high antibiotic and probiotic use may cause drastic changes in microbiome composition. The similarity of the managed African penguin cloaca microbiome to that of wild birds, and its dissimilarity to that of commercial poultry, implies that the exhibit and diet conditions at Georgia Aquarium resemble the wild rather than a domesticated environment. However, samples from wild penguins and other domesticated birds are necessary to validate this hypothesis.

## Conclusion

Our work identifies a baseline for evaluating the African penguin microbiome. While the cloaca and oral cavity of African penguins harbored distinct microbial communities that resembled those of other birds and marine vertebrates, respectively, all other body sites were similar to environmental samples, showing a tight connection with the managed care habitat. However, the cloaca microbiome of the African penguin did not resemble that of the domesticated chicken, possibly suggesting that the environment and diet in the managed care setting of Georgia Aquarium mimic wild conditions more closely than those applied in some domesticated bird facilities. Future studies would benefit from including wild animals to assess the influence of a managed care environment on the microbiomes of penguins and other species of conservation interest, and thereby potentially identify biomarkers of host and ecosystem health. Ultimately, this information could be used to inform management strategies in wild populations as well as those under managed care. In particular, the high percentage of taxa shared between environmental niches and the penguin external microbiome identifies exposure to surface and water microbiomes as a potentially significant vector for both commensal and pathogenic microbes. This connectivity highlights the necessity of maintaining clean exhibit conditions, as well as minimizing disturbance to microbial ecosystems influencing wild penguins. Future investigations of penguin microbiomes may inform and improve conservation efforts focused on mitigating the spread of disease.

### Supplementary Information

Supplementary Information.

## Data Availability

All raw data are publicly available at NCBI's SRA database under Bioproject PRJNA757721 and PRJNA757724.

## Abbreviations

PCR: Polymerase chain reaction
ASVs: Amplicon sequence variants
