# Supplementary material for: The microbiome of African penguins (Spheniscus demersus) under managed care resembles that of wild marine mammals and birds
Source: Sci Rep. 2023 Oct 4;13:16679. doi: 10.1038/s41598-023-43899-w (PMC10551019; doi:10.1038/s41598-023-43899-w)
Supplement: Supplementary file 1 — Supplementary Information. [file 41598_2023_43899_MOESM1_ESM.docx]

**Supplementary tables and figures for:**

**The microbiome of African penguins (*Spheniscus demersus*) under managed care resembles that of marine mammals and birds**

Ana G. Clavere Graciette, Lisa A. Hoopes, Tonya Clauss, Frank J. Stewart, Zoe A. Pratte

**Supplementary Table S1**. African penguin (*Spheniscus demersus)* metadata. Not all samples were acquired from all penguins, with "Yes" indicating the sample was taken from the oral cavity, cloaca, brood pouch, back skin, feathers, fecal, uropygial (preening) gland or leg skin. All penguins were residing at Georgia Aquarium at the time of sampling.

| **Penguin name** | **DOB** | **Age (yr)** | **Birth location** | **Months since molt** | **Nest partner** | **Date collected** | **Oral cavity** | **Cloaca** | **Brood pouch** | **Back skin** | **Feather** | **Fecal** | **Uropygial gland** | **Leg skin** |
| --- | --- | --- | --- | --- | --- | --- | --- | --- | --- | --- | --- | --- | --- | --- |
| B16004 | 6/2/15 | 4 | Other managed location | 10 | B15008 | 11/26/18 | Yes | Yes |  |  |  |  |  |  |
| B15002 | 1/15/16 | 3 | Georgia Aquarium | 12 | B15004 | 10/11/18 | Yes | Yes | Yes |  |  | Yes | Yes | Yes |
| B18001 | 1/9/18 | 1 | Georgia Aquarium | Unk | NA | 12/4/18 | Yes | Yes |  |  |  |  |  |  |
| B09009 | 10/14/04 | 15 | Other managed location | 15 | B10001 | 3/20/19 | Yes | Yes |  |  |  |  |  | Yes |
| B10010 | 3/10/09 | 10 | Other managed location | 13 | B09006 | 10/2/18 | Yes |  | Yes |  |  |  | Yes | Yes |
| B17028 | 12/29/17 | 1.5 | Georgia Aquarium | Unk | NA | 11/26/18 | Yes | Yes |  |  |  |  |  |  |
| B15004 | 1/16/15 | 4 | Georgia Aquarium | 12 | B15002 | 10/11/18 | Yes | Yes | Yes | Yes |  |  | Yes | Yes |
| B09002 | 1/30/15 | 4 | Other managed location | 14 | B09020 | 3/18/19 | Yes | Yes |  |  |  |  |  |  |
| B09013 | 6/18/09 | 9 | Other managed location | 13 | B09015 | 9/20/18 | Yes | Yes | Yes |  | Yes |  | Yes | Yes |
| B14002 | 1/9/14 | 4 | Georgia Aquarium | 11 | B13009 | 9/21/18 | Yes | Yes | Yes |  |  |  | Yes | Yes |
| B10019 | 7/7/09 | 9 | Other managed location | 14 | B10011 | 9/21/18 | Yes | Yes | Yes | Yes |  |  | Yes |  |
| B09015 | 6/20/09 | 10 | Other managed location | 6 | B09013 | 9/20/18 | Yes | Yes | Yes |  | Yes |  | Yes | Yes |
| B09020 | 3/11/09 | 10 | Other managed location | 11 | B09002 | 9/20/18 | Yes | Yes | Yes |  | Yes |  | Yes | Yes |
| B15006 | 2/28/15 | 4 | Georgia Aquarium | 13 | B13006 | 9/20/18 | Yes | Yes | Yes | Yes |  |  | Yes | Yes |
| B10011 | 4/13/09 | 10 | Other managed location | 13 | B10019 | 3/19/19 | Yes | Yes |  |  |  |  |  |  |
| B15009 | 3/3/15 | 4 | Georgia Aquarium | 4 | B13007 | 11/16/18 |  | Yes |  |  |  |  |  |  |
| B10006 | 10/9/08 | 10 | Other managed location | 13 | B09016 | 10/2/18 | Yes | Yes | Yes |  |  | Yes | Yes | Yes |
| B16001 | 1/9/16 | 3 | Georgia Aquarium | 9 | NA | 3/19/19 | Yes | Yes |  |  |  |  |  | Yes |
| B14001 | 1/1/14 | 5 | Georgia Aquarium | 13 | NA | 3/19/19 | Yes | Yes |  |  |  |  |  |  |
| B14004 | 1/14/14 | 5 | Georgia Aquarium | 15 | NA | 10/1/18 | Yes | Yes | Yes | Yes |  |  | Yes | Yes |
| B13002 | 1/15/13 | 6 | Georgia Aquarium | 8 | B14003 | 10/2/18 | Yes | Yes | Yes | Yes |  |  | Yes | Yes |
| B09018 | 3/6/87 | 32 | Other managed location | 13 | B09017 | 3/19/19 | Yes |  |  |  |  |  |  |  |
| B09003 | 2/2/05 | 19 | Other managed location | 13 | B09004 | 10/4/18 | Yes | Yes | Yes |  |  |  | Yes | Yes |
| B16002 | 1/19/16 | 3 | Georgia Aquarium | 5 | NA | 3/18/19 | Yes | Yes |  |  |  |  |  | Yes |
| B18004 | 1/28/18 | 1 | Georgia Aquarium | Unk | NA | 12/4/18 | Yes | Yes |  |  |  |  |  |  |
| B15008 | 3/3/15 | 4 | Georgia Aquarium | 9 | B16004 | 12/4/18 | Yes | Yes |  |  |  |  |  |  |
| B10009 | 3/2/09 | 9 | Other managed location | 15 | B10003 | 10/4/18 | Yes | Yes |  | Yes |  |  | Yes | Yes |
| B10003 | 10/12/08 | 10 | Other managed location | 9 | B10009 | 10/1/18 | Yes | Yes | Yes | Yes |  |  | Yes | Yes |
| B18002 | 1/12/18 | 1 | Georgia Aquarium | 4 | NA | 12/4/18 | Yes |  |  |  |  |  |  |  |
| B10017 | 7/10/09 | 10 | Other managed location | 14 | B10007 | 10/2/18 | Yes | Yes | Yes |  |  |  | Yes |  |
| B10007 | Unknown | Unk | Unknown | Unk | B10017 | 10/4/18 | Yes | Yes | Yes | Yes |  |  | Yes | Yes |
| B15010 | 3/5/15 | 4 | Georgia Aquarium | 13 | B15007 | 12/4/18 | Yes | Yes |  |  |  |  |  |  |
| B14007 | 2/6/14 | 5 | Georgia Aquarium | 13 | NA | 3/20/19 | Yes | Yes |  |  |  |  |  |  |
| B10022 | 4/8/09 | 10 | Other managed location | 14 | B09019 | 3/18/19 | Yes | Yes |  |  |  |  |  |  |
| B09019 | 10/23/08 | 10 | Other managed location | 14 | B10022 | 10/4/18 | Yes | Yes | Yes |  |  |  | Yes |  |
| B15007 | 3/2/15 | 4 | Georgia Aquarium | 13 | B15010 | 10/4/18 | Yes | Yes | Yes |  |  |  | Yes | Yes |

**Supplementary Table S2**. Pairwise Kruskal-Wallis tests based upon Shannon diversity indices comparing all African penguin body sites to one another and environmental samples. Global p value >0.0001. q-values represent p-values with a Benjamini-Hochberg FDR correction for multiple tests.

|  | **Group 1** | **Group 2** | **q-value** |
| --- | --- | --- | --- |
| Body sites vs Body sites | Cloaca | Uropygial (preening) gland | 0.004038462 |
|  | Cloaca | Oral cavity | 0.004038462 |
|  | Cloaca | Pouch | 0.004038462 |
|  | Cloaca | Back skin | 0.004038462 |
|  | Cloaca | Leg skin | 0.004038462 |
|  | Oral cavity | Pouch | 0.004038462 |
|  | Oral cavity | Back skin | 0.004038462 |
|  | Oral cavity | Leg skin | 0.004038462 |
|  | Uropygial (preening) gland | Oral cavity | 0.004038462 |
|  | Uropygial (preening) gland | Leg skin | 0.006363636 |
|  | Pouch | Back skin | 0.008076923 |
|  | Uropygial (preening) gland | Back skin | 0.008076923 |
|  | Uropygial (preening) gland | Pouch | 0.1850625 |
|  | Pouch | Leg skin | 0.232235294 |
| Cloaca vs Environment | Cloaca | Dry Rock | 0.018173077 |
|  | Cloaca | Feather | 0.015 |
|  | Cloaca | Fecal | 0.019811321 |
|  | Cloaca | Food | 0.004038462 |
|  | Cloaca | Dry guano | 0.012804878 |
|  | Cloaca | Nest | 0.004038462 |
|  | Cloaca | Shoreline | 0.004038462 |
|  | Cloaca | Wet Rocks | 0.004038462 |
|  | Cloaca | Water | 0.006363636 |
| Back Skin vs Environment | Back skin | Dry Rock | 0.316153846 |
|  | Back skin | Feather | 0.406617647 |
|  | Back skin | Fecal | 0.043032787 |
|  | Back skin | Food | 0.004038462 |
|  | Back skin | Dry guano | 0.166139241 |
|  | Back skin | Nest | 0.006363636 |
|  | Back skin | Shoreline | 0.0168 |
|  | Back skin | Wet Rocks | 0.044032258 |
|  | Back skin | Water | 0.037372881 |
| Leg Skin vs Environment | Leg skin | Dry Rock | 0.074307692 |
|  | Leg skin | Feather | 0.148636364 |
|  | Leg skin | Fecal | 0.014318182 |
|  | Leg skin | Food | 0.004038462 |
|  | Leg skin | Dry guano | 0.037372881 |
|  | Leg skin | Nest | 0.006363636 |
|  | Leg skin | Shoreline | 0.006363636 |
|  | Leg skin | Back skin | 0.006363636 |
|  | Leg skin | Wet Rocks | 0.042 |
|  | Leg skin | Water | 0.0105 |
| Oral Cavity vs Environment | Oral cavity | Dry Rock | 0.015 |
|  | Oral cavity | Feather | 0.004038462 |
|  | Oral cavity | Fecal | 0.006363636 |
|  | Oral cavity | Food | 0.004038462 |
|  | Oral cavity | Dry guano | 0.004038462 |
|  | Oral cavity | Nest | 0.004038462 |
|  | Oral cavity | Shoreline | 0.004038462 |
|  | Oral cavity | Wet Rocks | 0.008076923 |
|  | Oral cavity | Water | 0.008076923 |
| Brood Pouch vs Environment | Pouch | Dry Rock | 0.242215909 |
|  | Pouch | Feather | 0.23 |
|  | Pouch | Fecal | 0.015 |
|  | Pouch | Food | 0.004038462 |
|  | Pouch | Dry guano | 0.008076923 |
|  | Pouch | Nest | 0.004038462 |
|  | Pouch | Shoreline | 0.004038462 |
|  | Pouch | Wet Rocks | 0.014318182 |
|  | Pouch | Water | 0.015 |
| Uropygial (preening) gland vs. environment | Uropygial (preening) gland | Dry Rock | 0.591634615 |
|  | Uropygial (preening) gland | Feather | 0.185963855 |
|  | Uropygial (preening) gland | Fecal | 0.014318182 |
|  | Uropygial (preening) gland | Food | 0.004038462 |
|  | Uropygial (preening) gland | Dry guano | 0.032454545 |
|  | Uropygial (preening) gland | Nest | 0.004038462 |
|  | Uropygial (preening) gland | Shoreline | 0.004038462 |
|  | Uropygial (preening) gland | Wet Rocks | 0.015 |
|  | Uropygial (preening) gland | Water | 0.008076923 |

**Supplementary Table S3**. Pairwise PERMANOVA comparing all African penguin body sites to one another and environmental samples using weighted UniFrac distances. Global p-value >0.001. q-values represent p-values with a Benjamini-Hochberg FDR correction for multiple tests.

|  | **Group 1** | **Group 2** | **q-value** |
| --- | --- | --- | --- |
| Body sites vs. Body sites | Cloaca | Uropygial (preening) gland | 0.00404 |
|  | Cloaca | Oral cavity | 0.00404 |
|  | Cloaca | Brood pouch | 0.00404 |
|  | Cloaca | Back skin | 0.00404 |
|  | Cloaca | Leg skin | 0.00404 |
|  | Oral cavity | Brood pouch | 0.00404 |
|  | Oral cavity | Back skin | 0.00404 |
|  | Oral cavity | Leg skin | 0.00404 |
|  | Uropygial (preening) gland | Oral cavity | 0.00404 |
|  | Uropygial (preening) gland | Leg skin | 0.00636 |
|  | Back skin | Leg skin | 0.00636 |
|  | Uropygial (preening) gland | Back skin | 0.00808 |
|  | Brood pouch | Back skin | 0.00808 |
|  | Uropygial (preening) gland | Brood pouch | 0.18506 |
|  | Brood pouch | Leg skin | 0.23224 |
| Cloaca vs. Environment | Cloaca | Wet rocks | 0.00404 |
|  | Cloaca | Shoreline | 0.00404 |
|  | Cloaca | Food | 0.00404 |
|  | Cloaca | Nest | 0.00404 |
|  | Cloaca | Water | 0.00636 |
|  | Cloaca | Feces (dry) | 0.01280 |
|  | Cloaca | Feather | 0.01500 |
|  | Cloaca | Dry rocks | 0.01817 |
|  | Cloaca | Feces (fresh) | 0.01981 |
| Back skin vs. Environment | Back skin | Food | 0.00404 |
|  | Back skin | Nest | 0.00636 |
|  | Back skin | Shoreline | 0.01680 |
|  | Back skin | Water | 0.03737 |
|  | Back skin | Feces (fresh) | 0.04303 |
|  | Back skin | Wet rocks | 0.04403 |
|  | Back skin | Feces (dry) | 0.16614 |
|  | Back skin | Dry rocks | 0.31615 |
|  | Back skin | Feather | 0.40662 |
| Leg skin vs. Environment | Leg skin | Food | 0.00404 |
|  | Leg skin | Shoreline | 0.00636 |
|  | Leg skin | Nest | 0.00636 |
|  | Leg skin | Water | 0.01050 |
|  | Leg skin | Feces (fresh) | 0.01432 |
|  | Leg skin | Feces (dry) | 0.03737 |
|  | Leg skin | Wet rocks | 0.04200 |
|  | Leg skin | Dry rocks | 0.07431 |
|  | Leg skin | Feather | 0.14864 |
| Oral cavity vs. Environment | Oral cavity | Feather | 0.00404 |
|  | Oral cavity | Food | 0.00404 |
|  | Oral cavity | Nest | 0.00404 |
|  | Oral cavity | Shoreline | 0.00404 |
|  | Oral cavity | Feces (dry) | 0.00404 |
|  | Oral cavity | Feces (fresh) | 0.00636 |
|  | Oral cavity | Wet rocks | 0.00808 |
|  | Oral cavity | Water | 0.00808 |
|  | Oral cavity | Dry rocks | 0.01500 |
| Brood pouch vs. Environment | Brood pouch | Food | 0.00404 |
|  | Brood pouch | Nest | 0.00404 |
|  | Brood pouch | Shoreline | 0.00404 |
|  | Brood pouch | Feces (dry) | 0.00808 |
|  | Brood pouch | Wet rocks | 0.01432 |
|  | Brood pouch | Feces (fresh) | 0.01500 |
|  | Brood pouch | Water | 0.01500 |
|  | Brood pouch | Feather | 0.23000 |
|  | Brood pouch | Dry rocks | 0.24222 |
| Uropygical (preening) gland vs. Environment | Uropygial (preening) gland | Shoreline | 0.00404 |
|  | Uropygial (preening) gland | Nest | 0.00404 |
|  | Uropygial (preening) gland | Food | 0.00404 |
|  | Uropygial (preening) gland | Water | 0.00808 |
|  | Uropygial (preening) gland | Feces (fresh) | 0.01432 |
|  | Uropygial (preening) gland | Wet rocks | 0.01500 |
|  | Uropygial (preening) gland | Feces (dry) | 0.03245 |
|  | Uropygial (preening) gland | Feather | 0.18596 |
|  | Uropygial (preening) gland | Dry rocks | 0.59163 |

**Supplementary Table S4.** Number of ASVs shared between each African penguin (*Spheniscus demersus*) body site and the environment, and of the number of unique ASVs for each penguin body site, as well as their associated proportion.

| Body site | Number of ASVs shared with environment | Proportion of ASVs shared with the environment | Number of unique ASVs | Proportion of unique ASVs |
| --- | --- | --- | --- | --- |
| Cloaca | 336 | 41.227% | 182 | 22.331% |
| Oral cavity | 291 | 44.769% | 120 | 18.462% |
| Brood pouch | 709 | 61.921% | 103 | 8.996% |
| Uropygial (preening) gland | 674 | 63.168% | 75 | 7.029% |
| Back skin | 511 | 62.931% | 52 | 6.404% |
| Leg skin | 724 | 64.528% | 92 | 8.200% |

**Supplementary Table S5**. Pairwise Kruskal-Wallis tests based upon Shannon diversity indices comparing the oral microbiome of the African penguin to various vertebrate hosts including birds, reptiles, and mammals. Global p-value >0.0001. q-values represent p-values with a Benjamini-Hochberg FDR correction for multiple tests.

| **Group 1** | **Group 2** | **q-value** |
| --- | --- | --- |
| African penguin | Human | 0.00001 |
| African penguin | Cooper’s hawk | 0.00034 |
| African penguin | Sea lion | 0.00034 |
| African penguin | Bottlenose dolphin | 0.00081 |
| African penguin | Prairie rattlesnake | 0.00438 |
| African penguin | Komodo dragon | 0.01400 |
| African penguin | Great tit | 0.91924 |

**Supplementary Table S6**. Pairwise PERMANOVA comparing the oral microbiomes of the African penguin to various vertebrate hosts including birds, reptiles, and mammals using weighted UniFrac distances. Global p-value = 0.001. q-values represent pairwise p-values with a Benjamini-Hochberg FDR correction for multiple tests.

| **Group 1** | **Group 2** | **q-value** |
| --- | --- | --- |
| African penguin | Bottlenose dolphin | 0.00138 |
| African penguin | Great tit | 0.00138 |
| African penguin | Cooper’s hawk | 0.00138 |
| African penguin | Human | 0.00138 |
| African penguin | Komodo dragon | 0.00138 |
| African penguin | Prairie rattlesnake | 0.00138 |
| African penguin | Sea lion | 0.00138 |

**Supplementary Table S7**. Pairwise Kruskal-Wallis tests based upon Shannon diversity indices comparing the fecal/cloaca microbiomes of African penguins compared to various vertebrate hosts including birds, reptiles, and mammals. Global p-value >0.0001. q-values represent pairwise p-values with a Benjamini-Hochberg FDR correction for multiple tests.

| **Group 1** | **Group 2** | **q-value** |
| --- | --- | --- |
| African penguin | Bottlenose dolphin | 0.00001 |
| African penguin | Komodo dragon | 0.00046 |
| African penguin | Crocodile lizard | 0.01138 |
| African penguin | Sea lion | 0.01751 |
| African penguin | Barn swallow | 0.57746 |
| African penguin | Great tit | 0.57746 |
| African penguin | Human | 0.65115 |
| African penguin | Chicken | 0.75042 |

**Supplementary Table S8**. Pairwise PERMANOVA comparing the fecal/cloaca microbiomes of African penguins compared to various vertebrate hosts including birds, reptiles, and mammals using weighted UniFrac distances. Global p-value =0.001. q-values represent p-values with a Benjamini-Hochberg FDR correction for multiple tests.

| **Group 1** | **Group 2** | **q-value** |
| --- | --- | --- |
| African penguin | Barn swallow | 0.00105 |
| African penguin | Chicken | 0.00105 |
| African penguin | Crocodile lizard | 0.00105 |
| African penguin | Bottlenose dolphin | 0.00105 |
| African penguin | Great tit | 0.00105 |
| African penguin | Human | 0.00105 |
| African penguin | Komodo dragon | 0.00105 |
| African penguin | Sea lion | 0.00105 |

**Supplementary Figures**

**Supplementary Figure S1:** Heatmap indicating the average relative abundance of phyla composing the fecal or cloaca microbiome of various wild and managed vertebrate hosts (birds, reptiles, and mammals).

**Supplementary Figure S2:** Heatmap indicating the average relative abundance of genera composing the fecal or cloaca microbiome of various wild and managed vertebrate hosts (birds, reptiles, and mammals). Only the 10 most abundant genera for each host species are included- the remainder are not shown. Not all taxa could be classified to the genus level.

**Supplementary Figure S3.** Standard box plots representing differences in alpha diversity (observed ASVs and Shannon diversity index) for different African penguin body sites and environmental samples from their exhibit. Significant differences for the Shannon diversity index are given in Supplementary Table S2.

**Supplementary Figure S4.** Heat map indicating the average relative abundance of phyla composing the penguin (*Spheniscus demersus*) microbiome at different body sites and environmental samples collected from the penguin exhibit at Georgia Aquarium.

**Supplementary Figure S5.** Heat map indicating the average relative abundance of genera composing the penguin (*Spheniscus demersus*) microbiome at different body sites and environmental samples collected from the penguin exhibit at Georgia Aquarium. Only the 10 most abundant genera for each category are included- the remainder are not shown. Not all taxa could be classified to the genus level.

**Supplementary Figure S6.** Heat map indicating the average relative abundance of phyla composing the oral microbiome of various vertebrate hosts (birds, reptiles, and mammals).

**Supplementary Figure S7.** Heat map indicating the average relative abundance of genera composing the oral microbiome of various vertebrate hosts (birds, reptiles, and mammals). Only the 10 most abundant genera for each host species are included- the remainder are not shown. Not all taxa could be classified to the genus level.
